# Efficacy and safety of 5-hydroxytryptamine-3 (5-HT3) receptor antagonists in augmentation with selective serotonin reuptake inhibitors (SSRIs) in the treatment of moderate to severe obsessive–compulsive disorder: a systematic review and meta-analysis of randomized clinical trials

**DOI:** 10.1038/s41598-023-47931-x

**Published:** 2023-11-27

**Authors:** Negin Eissazade, Hesam Mosavari, Shayan Eghdami, Mahsa Boroon, Faria Ashrafi, Mohammadreza Shalbafan

**Affiliations:** 1 Student Research Committee, School of Medicine, Iran University of Medical Sciences, Tehran, Iran; 2 Department of Surgery, General Surgery Research Center, School of Medicine, Rasool-E Akram Hospital, Iran University of Medical Sciences, Tehran, Iran; 3 Cellular and Molecular Research Center, Iran University of Medical Sciences, Tehran, Iran; 4 Department of Psychiatry, Imam Hossein Hospital, School of Medicine, Alborz University of Medical Sciences, Karaj, Iran; 5 Brain and Cognition Clinic, Institute for Cognitive Sciences Studies, Tehran, Iran; 6 Mental Health Research Center, Psychosocial Health Research Institute (PHRI), Department of Psychiatry, School of Medicine, Iran University of Medical Sciences, Tehran, Iran

**Keywords:** Obsessive compulsive disorder, Clinical pharmacology

## Abstract

Obsessive–compulsive disorder (OCD) is the fourth most common mental disorder, and selective serotonin reuptake inhibitors (SSRIs) are the cornerstone of its pharmacological treatment. About 40–60% of the cases are treatment-refractory, and this makes searching for second-line treatment necessary. 5-Hydroxytryptamine-3 (5-HT3) antagonists are among the many medications that have been used in augmentation with SSRIs. In this systematic review and meta-analysis, we assessed the efficacy and safety of 5-HT3 receptor antagonists in augmentation with SSRIs in treating moderate to severe OCD. We searched PubMed, Web of Science, Scopus, Cochrane library, and Google Scholar for relevant trials published up to December 2022. The effect size was the mean difference in Yale-Brown obsessive compulsive scale (Y-BOCS) scores before and after receiving 5-HT3 receptor antagonist drugs in augmentation with SSRIs in moderate to severe OCD patients. We included 6 randomized-controlled trails (RCTs) with 334 patients assessing the effect of the augmentation of SSRIs with ondansetron, granisetron, and tropisetron on treating moderate to severe OCD. Our results were in favor of the experimental group in total (Z = 8.37, P < 0.00001), in the compulsion subgroup (Z = 5.22, P < 0.00001), and in the obsession subgroup (Z = 8.33, P < 0.00001). They are well-tolerated, and have mild side effects and do not result in withdrawal. Augmentation of 5-HT3 antagonists with SSRIs can be beneficial in treating moderate to severe OCD. Further multi-center trials under adequate conditions in longer periods are needed to help come up with a comprehensive action plan.

## Introduction

Obsessive–compulsive disorder (OCD) affects approximately 2% of the general population, and it is the fourth most common mental disorder after depression, alcohol/substance use disorders, and social phobia^1^. OCD is characterized by the presence of obsessions, compulsions, or both. Obsessions are recurrent intrusive thoughts, urges, or images that the patient tries to ignore or suppress. They commonly present as fear of contamination or losing control or harm, sexual fears, religious fears, and perfectionism. Subsequently, compulsions occur to prevent or reduce the anxiety and discomfort caused by obsessions. They may present as repetitive behaviors (e.g., washing and cleaning, checking, and ordering) or mental acts (e.g., praying and counting)^2^. As OCD causes significant functional impairment and leads to income loss and decreased quality of life, World Health Organization (WHO) lists it as one of the ten most disabling conditions^1^.

SSRIs (fluoxetine, fluvoxamine, paroxetine, and sertraline) and the SRI, clomipramine, are the first-line FDA-approved medications for the treatment of OCD^3^. However, 40–60% of the cases are treatment-refractory^4^. Not achieving adequate symptom relief after the first-line treatment, highlights the importance of second-line and adjuvant therapy.

The exact etiopathology of OCD is still unknown. Acceptable response to treatment with SSRIs suggested a possible role for serotonin, which may be through serotonin–dopamine interactions^5^. Glutamate and GABA play an essential role in the development of normal cortico-striatal-thalamo-cortical (CSTC) pathway. Subsequently, their dysregulation causes dysfunctional development of CSTC, which may be the underlying cause of OCD^6^. In patients with OCD, glutamate and its products are elevated in the basal ganglia and reduced in the anterior cingulate cortex. As serotonin receptors are involved in the cortical and thalamic glutamatergic input to basolateral amygdala, a possible role for glutamate through serotonin receptors is suggested^7^. Moreover, decreased number of regulatory T-cells in moderate to severe Tourette syndrome/OCD suggested OCD’s association with inflammation and immune dysregulation^8^.

Thus far, different medications have been used in augmentation with SSRIs in the treatment of OCD. World Federation of Societies of Biological Psychiatry (WFSBP) guidelines have stated that augmentation of the antipsychotics risperidone, haloperidol, olanzapine, or quetiapine with an SSRI is more effective than monotherapy with SSRIs^9^. Other atypical antipsychotics, such as aripiprazole^10^, may also be beneficial. Glutamatergic medications such as memantine^11^, *N*-acetyl cysteine (NAC)^12^, minocycline^13^, l-carnosine^14^, and riluzole^15^, have also been proven tolerable and effective^16^. In addition to its glutamergic role, minocycline is an antioxidant and anti-inflammatory antibiotic that regulates nitric oxide, tumor necrosis factor-α (TNF-α), and interleukin-1β^17^. Topiramate^18^ and lamotrigine^19^ have shown some efficacy through GABA and glutamate receptors. SSRIs’ augmentation with celecoxib, a cyclooxygenase-2 (COX-2) inhibitor and non-steroidal anti-inflammatory drug, has also been reported to be tolerable and effective^20–22^. As 5-hydroxytryptophan is the intermediate metabolite of l-tryptophan (the amino acid used to produce serotonin), it is a promising new candidate for adjuvant therapy in treating OCD^23^.

Existing preclinical evidence supports the possible role of the 5-HT3 receptor antagonists in treating psychiatric disorders^24,25^. 5-HT3 receptor antagonists include ondansetron, granisetron, tropisetron, and palonosetron. They are safe and effective antiemetic agents, mainly used for postsurgical and post-chemotherapy nausea and vomiting^26^. They block serotonin both peripherally and centrally^27^. 5-HT3 stimulation enhances dopaminergic activity and the release of serotonin, norepinephrine, and acetylcholine^28^. A systematic review and meta-analysis conducted in 2017 indicated some utility of 5-HT3 antagonists in treating OCD^29^.

Considering the newly reported RCTs of augmentation of the medications of this group with SSRIs for the treatment of patients with moderate to severe OCD, this study aimed to update the systematic review and meta-analysis of the efficacy and safety of 5-HT3 receptor antagonists in augmentation with SSRIs in treating moderate to severe OCD.

## Methods

We conducted this systematic review following the Preferred Reporting Items for Systematic Reviews and Meta-Analyses for Protocols guidelines (PRISMA)^30^.

### Eligibility criteria

Studies were included if: (1) they described participants older than 18 years of age who were diagnosed with OCD according to the Diagnostic and Statistical Manual of Mental Disorders (DSM), fourth or fifth edition; (2) the Yale-Brown Obsessive Compulsive Scale (Y-BOCS)^31^ was used as a measure of OCD symptoms severity before and following treatment with 5-HT3 receptor antagonist drugs. The Y-BOCS is a 10-item scale used to estimate the severity of OCD symptoms. This scale has a total score of 0 to 40, and higher scores indicate more severe symptoms; (3) participants had moderate to severe symptoms of OCD (Y-BOCS score > 16)^31^; (4) the trial duration was eight weeks or more.

### Information sources and search procedure

The electronic databases of PubMed, Web of Science, Scopus, Cochrane Library, Google Scholar and ProQuest (Grey Literature) were searched for relevant trials based on the search strategies described in the protocol (Supplementary Appendix 1) to gather the body of evidence available from original articles published up to December 2022 in English. One of the authors (HM) conducted an electronic database search, imported citations from all databases into an Endnote library (version 20, Thomson Reuters, USA), and removed duplicate citations using the “Find Duplicates” feature of Endnote software.

### Outcome measures

Our primary outcome measure (effect size) was the mean difference in Y-BOCS rating before and after receiving 5-HT3 receptor antagonist drugs in patients with OCD who were under treatment with SSRIs.

### Study selection

Two authors (HM and NE) independently screened the titles and abstracts of studies initially selected to eliminate reminders of duplicate citations and those that were irrelevant, then retrieved the remainder of the citations’ full texts for further screening. Two authors (HM and NE) read full texts of articles carefully and independently included eligible articles based on the abovementioned inclusion criteria. Protocols, design and development papers, and opinion pieces were excluded. Disagreements between the two authors were resolved by discussion or consultation with a third author (MS). To identify other relevant papers, we checked the references of these articles. We contacted the authors of ongoing clinical trials to identify any relevant forthcoming or unpublished studies. The full texts of the final selected studies were examined for quality assessment, data collection, and analysis.

### Data extraction and quality assessment

After the initial screening, the full texts were reviewed by two independent researchers to include eligible articles according to the inclusion criteria.

Detailed data extraction was performed based on the pre-designed data extraction forms. Extracted information included the study design, name and email address of the corresponding author, participants’ characteristics, interventions, and outcomes.

Two reviewers independently evaluated studies for risk of bias in six domains of random sequence generation (selection bias for controlled trials), allocation concealment (selection bias for controlled trials), blinding of participants and personnel (performance bias), blinding of outcome assessment (detection bias), incomplete outcome data (attrition bias) and selective reporting (reporting bias) according to the Cochrane “Risk of bias” tool and attributed each domain to be of “low risk”, “high risk”, or “some concerns” of bias for each article^32^. Any disagreement between the two authors regarding the quality assessment was resolved through discussion or consultation with the third author.

### Evidence synthesis

We developed an evidence synthesis of the findings of the included studies using systematic approaches such as textual descriptions, tabulation, and transforming data into a common rubric using Review Manager (RevMan) (Version 5.4. The Cochrane Collaboration, 2020). For missing data, we used statistical methods to calculate missing data using data presented in the articles. We used Wan’s method to estimate the mean and standard deviation from the sample size, median, range, and interquartile range^33^. We considered participants with missing data as non-abstainers.

We performed a meta-analysis Review Manager and estimated a weighted average treatment effect across studies. Heterogeneity across studies was evaluated by the chi-square statistic and calculation of I^2^ (defined as I^2^ > 40% or chi-square statistic P < 0.1). A random-effects model was used in case of significant statistical heterogeneity. Moreover, we applied a subgroup analysis in the case of clinical heterogeneity.

We expressed meta-analysis results in risk ratio (RR) with a 95% confidence interval (CI) for dichotomous outcomes. For each outcome, we used data from the most extended follow-up period reported in the study.

### Ethics approval and consent to participate

The study was approved by the ethics committee of Iran University of Medical Sciences institutional review board (IRB: IR.IUMS.REC.1401.695).

## Results

### Description of included studies

**Figure 1:**
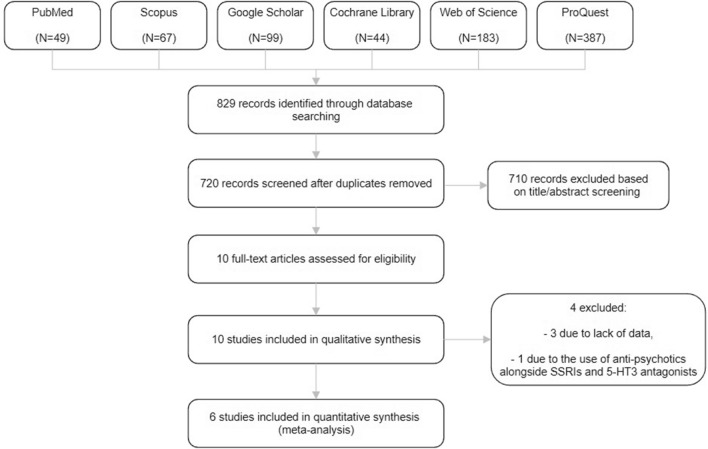
Flowchart of study selection for systematic review and meta-analysis of the effect of 5-HT3 antagonists in the treatment of moderate to severe obsessive–compulsive disorder.

**Figure 2:**
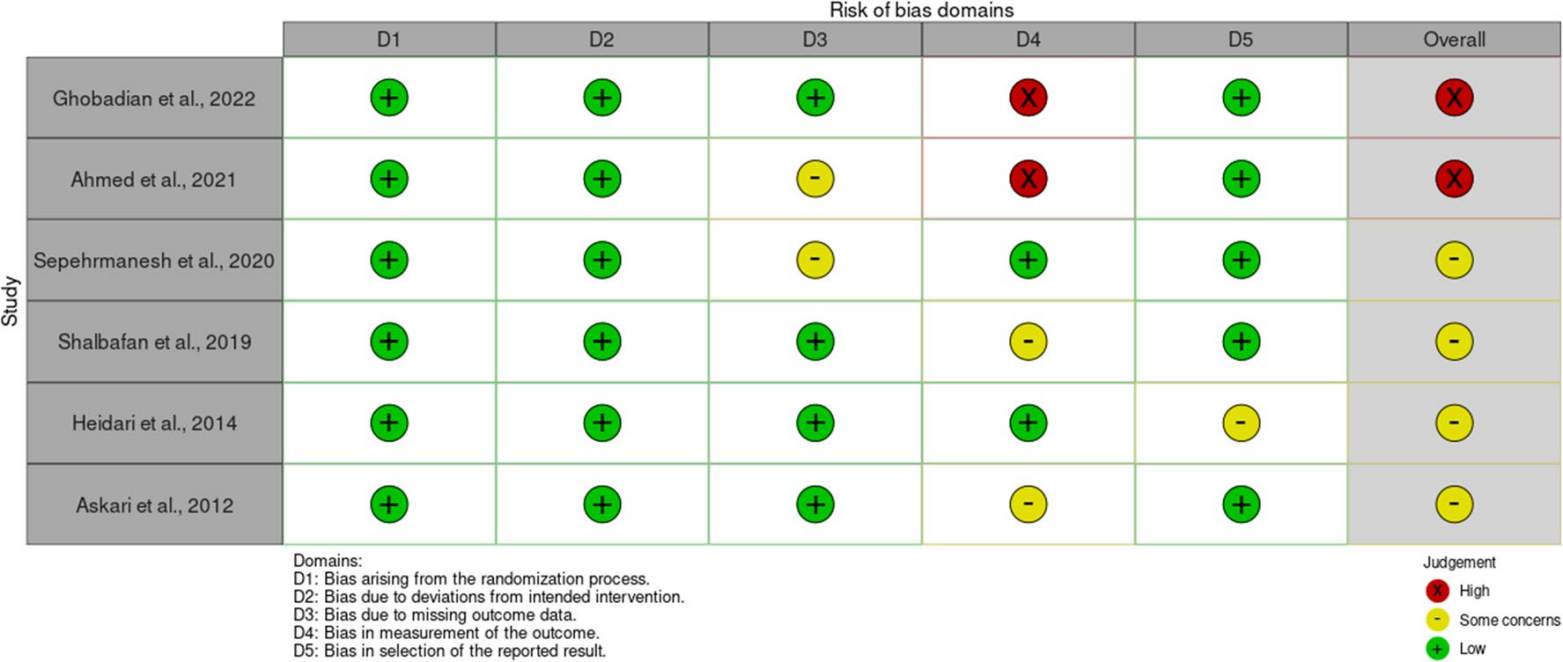
Risk of bias for the studies included in meta-analysis considering.

The literature search provided a total of 829 potentially relevant publications which were screened for eligibility. After screening titles and abstracts, ten articles entered full-text screening. Thereafter, 6 RCTs^34–39^ with 334 patients with OCD met the inclusion criteria and were included in this systematic review (Fig. 1). These studies are presented in Table 1. The overall quality of studies was fair (Fig. 2). All six studies have recommended the augmentation of 5-HT3 antagonists with SSRIs in treating moderate to severe OCD.

**Table 1:** Summary of clinical trials investigating the effect of 5-HT3 receptor antagonists in treating moderate to severe obsessive–compulsive disorder included in meta-analysis.

Study (first author, year of study)	Participants		Intervention	Follow-up time	Change of mean differences of Y-BOCS total score (baseline-endpoint), 95% CI experimental group vs. control group	Adverse effects
	Experimental group	Placebo group				
Ghobadian et al. (2022)^34^	25	20	Granisetron 1mg BD/placebo + sertraline (100 mg/day for 4 weeks, 200 mg/day)	10 weeks	11.2 (6.65–15.74) vs. 8.4 (4.78–12.01), P = 0.001	No significant difference between the two groups
Ahmed et al. (2021)^35^	34	36	Ondansetron 2 mg BD/placebo + fluoxetine (20 mg/day for 1 week, 40–80 mg/day)	12 weeks	16.97 (14.89‑19.04) vs. 11.72 (9.73‑13.71), P = 0.001	No significant difference between the two groups
Sepehrmanesh et al. (2020)^36^	20	20	Ondansetron 8 mg daily/placebo + SSRIs (not specified)	12 weeks	9.2 (6.91–11.49) vs. 4.5 (1.47–7.53), P = 0.02	Good tolerability
Shalbafan et al. (2019)^37^	48	48	Tropisetron 5 mg BD/placebo + fluvoxamine100 mg/daily 4 weeks, 200 mg/daily)	10 weeks	12.69 (10.93–14.44) vs. 9.83 (7.73–11.94), P < 0.001	No significant difference between the two groups
Heidari et al. (2014)^38^	22	22	Ondansetron 4 mg BD/placebo + fluvoxamine (100mg/day for 4 weeks, 200 mg)	8 weeks	14.4 (9.9–18.19) vs. 8.9 (1.2–16.6), P = 0.006	No significant difference between the two groups
Askari et al. (2012)^39^	19	20	Granisetron 1 mg BD/placebo + fluvoxamine 100 mg/day for 4 weeks, 200 mg)	8 weeks	16.8 (10.9–22.7) vs. 9.9 (0.8–19), P = 0.008	No significant difference between the two groups

We measured the mean difference (MD) in Y-BOCS ratings before and after the pharmacological intervention in experimental and control groups, in the compulsion and obsession subgroups, and overall.

**Figure 3:**
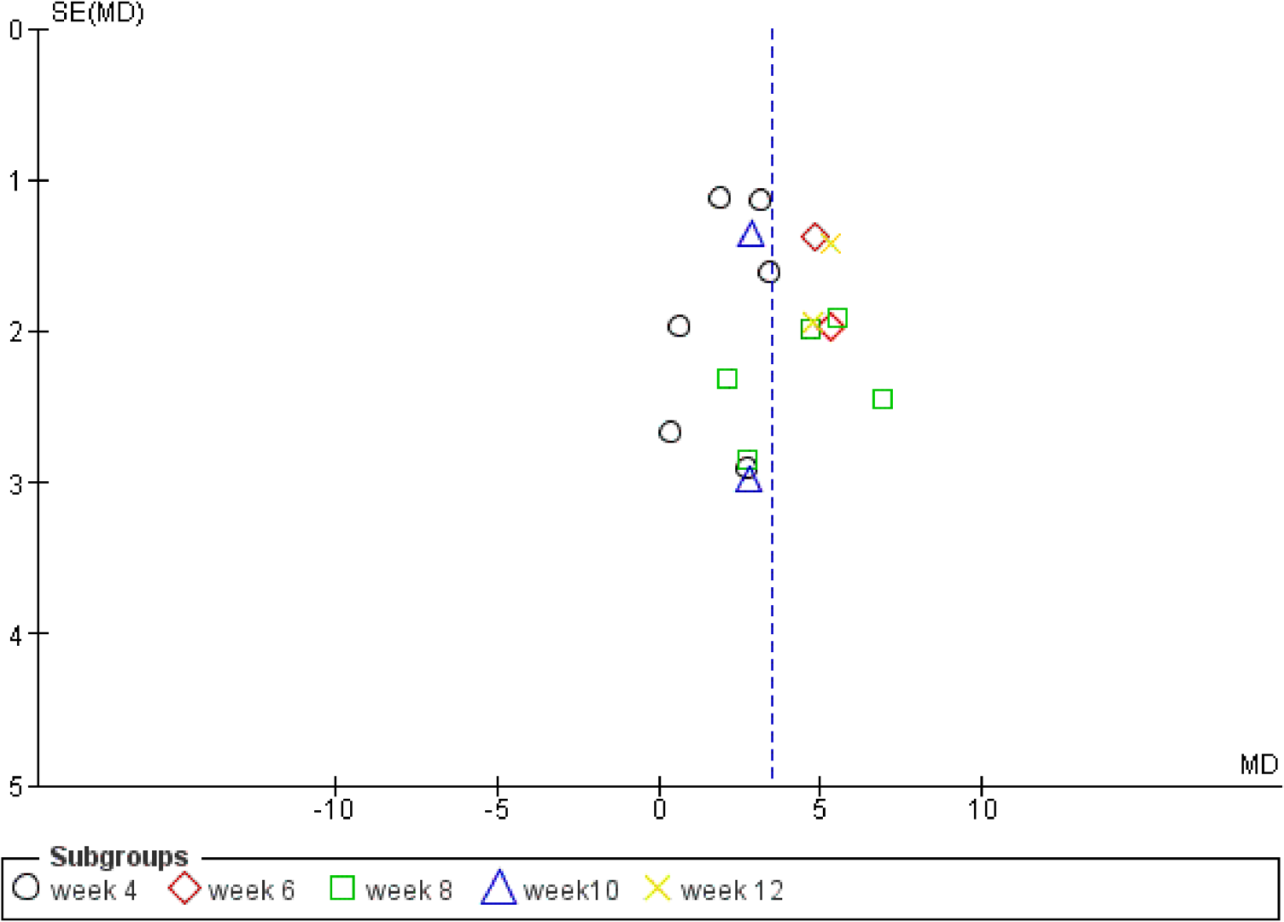
Funnel plot of Y-BOCS score between patients with moderate to severe obsessive–compulsive disorder in experimental groups and control groups in week 4, 6, 8, and 10 in total.

**Figure 4:**
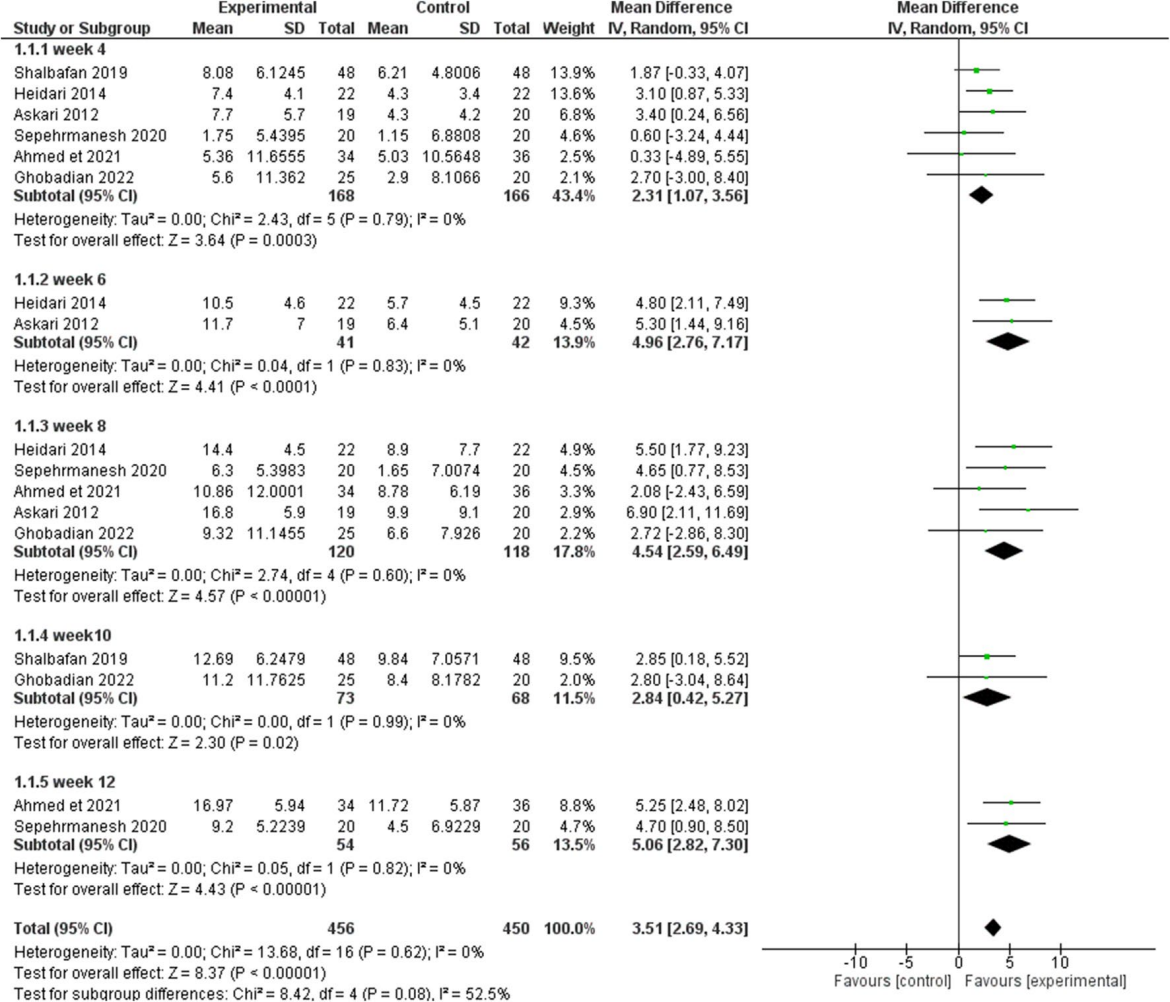
Comparison of Y-BOCS score between patients with moderate to severe obsessive–compulsive disorder in experimental groups and control groups in week 4, 6, 8, and 10 in total.

**Figure 5:**
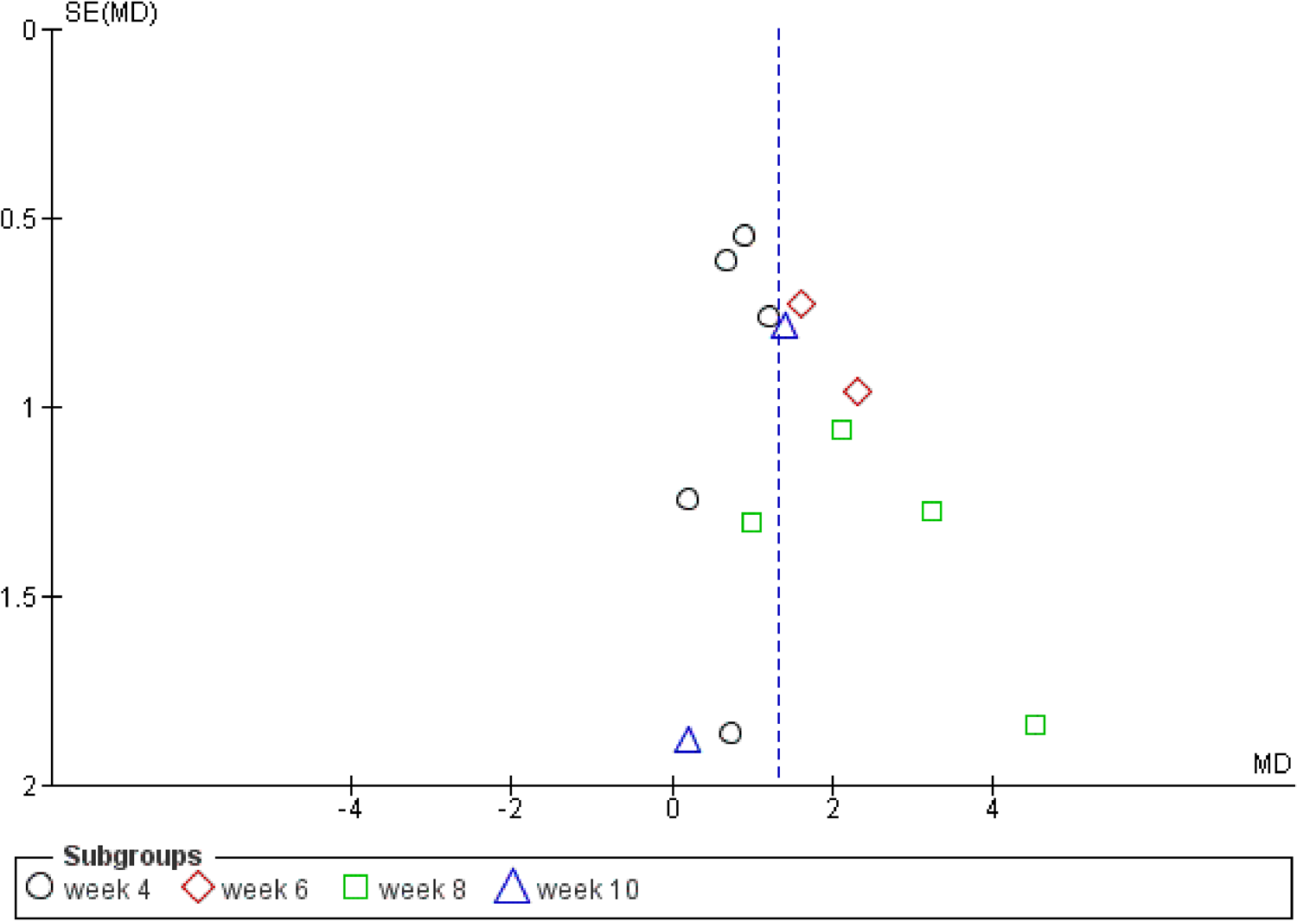
Funnel plot of Y-BOCS score between patients with moderate to severe obsessive–compulsive disorder in experimental groups and control groups in week 4, 6, 8, and 10 in the compulsion subgroup.

**Figure 6:**
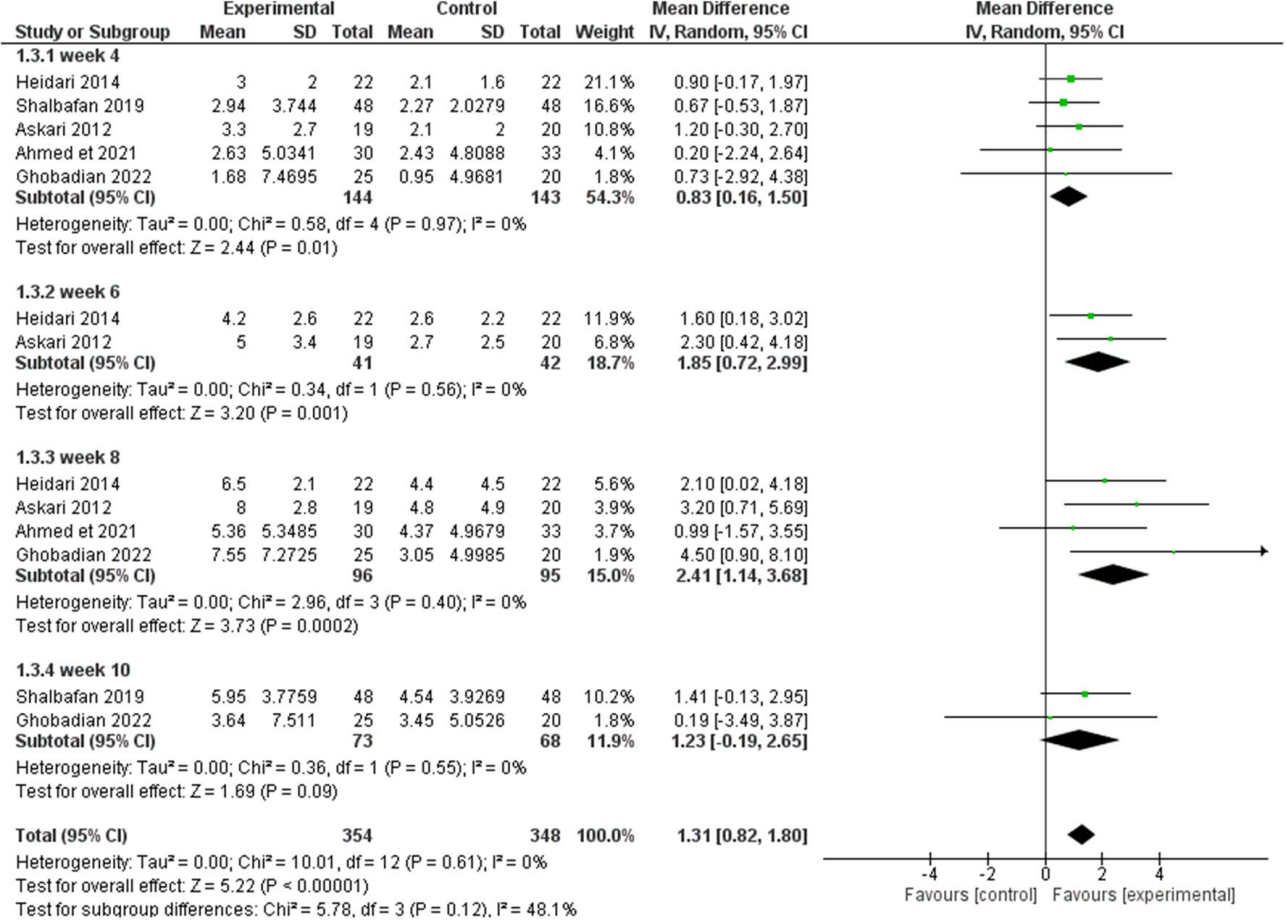
Comparison of Y-BOCS score between patients with moderate to severe obsessive–compulsive disorder in experimental groups and control groups in week 4, 6, 8, and 10 in the compulsion subgroup.

**Figure 7:**
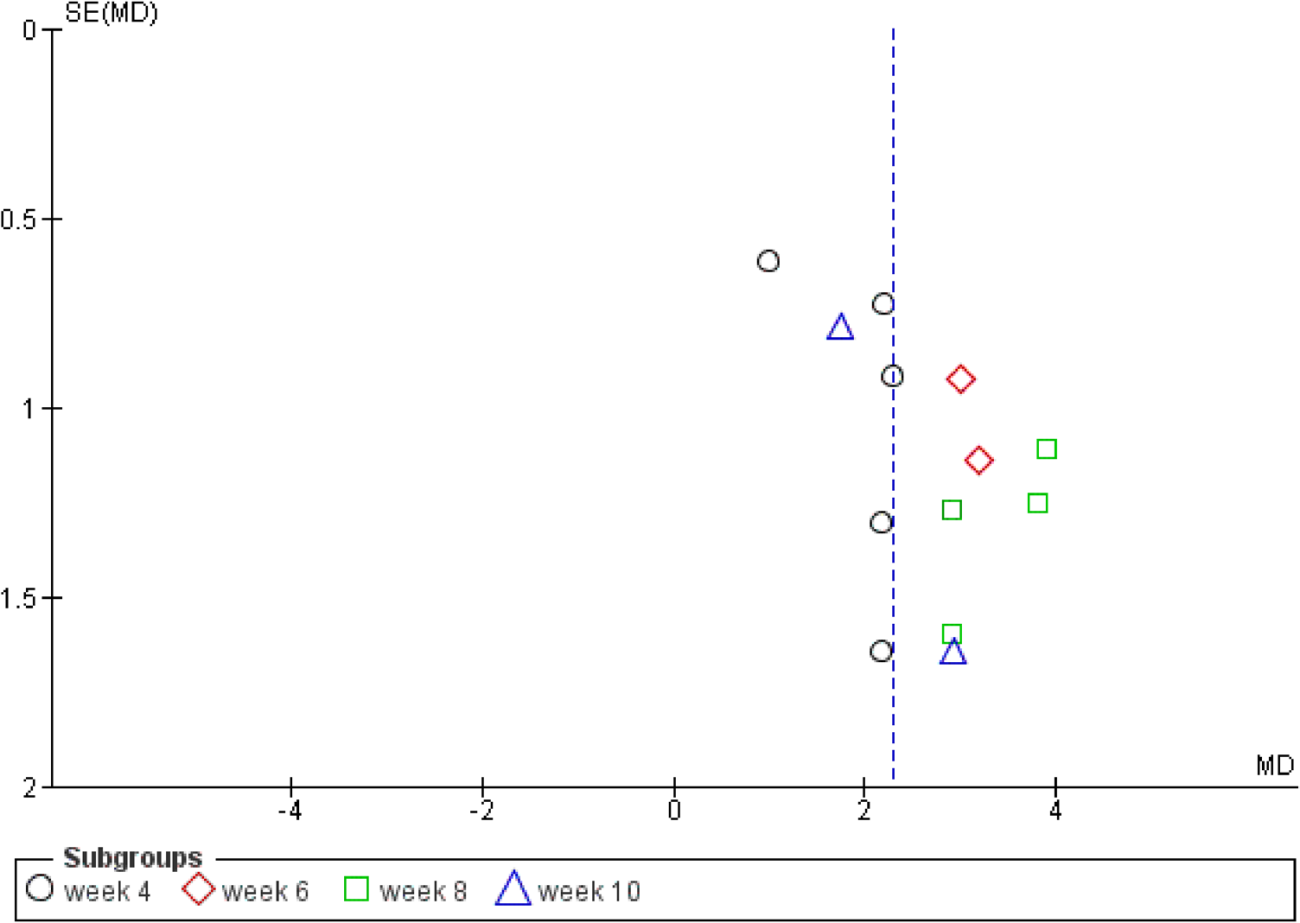
Funnel plot of Y-BOCS score between patients with moderate to severe obsessive–compulsive disorder in experimental groups and control groups in week 4, 6, 8, and 10 in the obsession subgroup.

**Figure 8:**
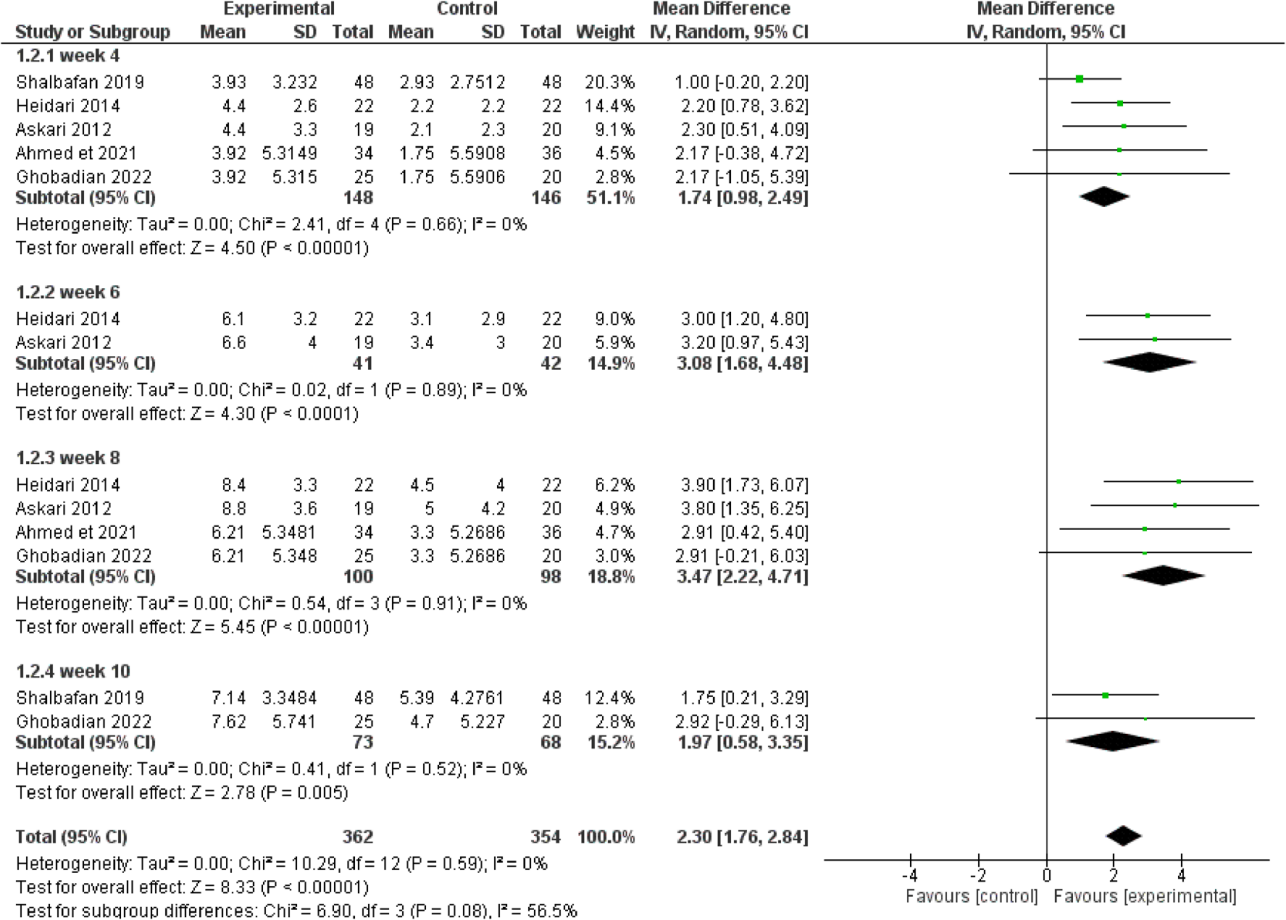
Comparison of Y-BOCS score between patients with moderate to severe obsessive–compulsive disorder in experimental groups and control groups in week 4, 6, 8, and 10 in the obsession subgroup.

Results were in favor of the experimental group in total (Z = 8.37, P < 0.00001) (Figs. 3, 4), in the compulsion subgroup (Z = 5.22, P < 0.00001) (Figs. 5, 6), and in the obsession subgroup (Z = 8.33, P < 0.00001) (Figs. 7, 8) confirming the hypothesis that 5-HT3 antagonists can be effective in augmentation with SSRIs in the treatment of moderate to severe OCD.

### Ondansetron

Ondansetron has an excellent effect against nausea and vomiting. In the articles published by Heidari et al., Sepehrmanesh et al. and Ahmed et al., the effect of ondansetron in augmentation with SSRIs was investigated and the three articles reported significant beneficial effect^35,36,38^. Heidari et al. and Ahmed et al. reported that the frequency and severity of adverse events were not significantly different between the two groups, which were headache, dry mouth, constipation, dizziness, muscle cramp, insomnia, diarrhea, and nervousness. Sepehrmanesh et al. reported that ondansetron was well-tolerated.

Pallantia et al. and Soltani et al. reported that patients with OCD may benefit from augmentation of low-dose ondansetron^40–42^. However, these three articles were excluded from meta-analysis due to insufficient data. Pallantia et al. reported that adverse effects were mild to moderate and included decreased appetite and headache, and a mild increase in anxiety levels in one patient for only one night. Pallantia et al. reported that adverse events were mild and withdrawal, and included headache, dizziness and constipation. Soltani et al. reported that one patient in ondansetron group left the study due to severe headaches in ondansetron group, and one patient in placebo group experienced insomnia which was not significant.

Sharafkhah et al.^43^ assessed the efficacy and tolerability of ondansetron vs. granisetron in patients with treatment-resistant OCD. However, their study was excluded from our meta-analysis as they had augmented 5-HT3 antagonists with antipsychotics and SSRIs. This study confirmed the beneficence of both medications, with the superiority of ondansetron, as the ondansetron group had a significantly higher rate of complete response and no relapse was observed. Side effects were mild (diarrhea in the ondansetron group, and dry mouth and nervousness in the placebo group), and no withdrawal symptom was reported.

### Granisetron

Granisetron is a highly selective 5-HT3 receptor antagonist. As investigated by Askari et al. and Ghobadian et al., it can be beneficial for moderate to severe OCD^34,39^. The frequency of adverse events was not significantly different between the two groups. Askari et al. reported mild and transient drowsiness, dry mouth, constipation, dizziness, sweating, nausea, muscle tension, decreased appetite, nervousness, itching, fatigue, diarrhea, and sexual dysfunction. Moreover, Ghobadian et al. reported mild adverse effects without resulting in withdrawal: pain, headache, diarrhea, constipation, decreased libido, decreased appetite, lightheadedness, tremor, skin lesions, motor tic, palpitation, insomnia, itching, restlessness, and vomiting.

Sharafkhah et al.^43^ reported that relapse occurred in 7.31% of the patients in the granisetron group. Side effects were mild (constipation and headache) and were not statistically different from the ondansetron group.

### Tropisetron

Tropisetron’s effect on OCD has only been investigated in one study by Shalbafan et al.^37^ in which they reported that tropisetron can have therapeutic effects on OCD. Furthermore, the frequency of adverse events was not significantly different between the two groups: headache, diarrhea, increased appetite, dizziness, insomnia, nausea, sedation, and abdominal pain.

### Palonosetron

Thus far, the effect of palonosetron augmentation with SSRIs in treating moderate to severe OCD has not been investigated.

## Discussion

The role of various neurotransmitters (serotonin, norepinephrine, acetylcholine, and dopamine) in the physiopathology of OCD has been discussed before, which has opened many doors for different categories of medications in treating OCD, such as SSRIs, clomipramine, glutamergics, and anti-inflammatories. Inadequate response to first-line treatment, necessitates searching for efficacious second-line treatment.

5-HT3 receptors are the ionotropic 5-HT receptors. They are primarily localized in the limbic structures (e.g., amygdala, hippocampus, nucleus accumbens and striatum) and only a low density of 5-HT3 receptors exists in the cortex^44^. 5-HT3 stimulation enhances dopaminergic activity and the release of serotonin, norepinephrine, and acetylcholine^5,28^. Selective serotonin receptor (5-HT3) antagonists block serotonin both peripherally on vagal nerve terminals in the gastrointestinal system and centrally in the chemoreceptor trigger zone in the hindbrain in particular in the dorsal motor nucleus of the vagus nerve, in the nucleus of the tractus solitaries and the area postrema of the fourth ventricle, resulting in powerful antiemetic effects^27^. They do not have substantial sedative effects, minimizing the risk of abuse, dependence, tolerance, or withdrawal^45^. In addition, SSRIs commonly produce digestive side effects such as nausea and vomiting, diarrhea, and decreased appetite. These side effects are determined by the increase in serotonin availability at 5-HT3 receptors. 5-HT3 antagonists antagonize these effects and make them more tolerable^46^.

Based on the results of this systematic review and meta-analysis, ondansetron, granisetron, and tropisetron have beneficial effects in treating moderate to severe OCD. They are well-tolerated, have mild side effects and do not result in withdrawal.

The clinical trials that were included in our study had limitations as being single-centered, having small sample sizes, having short follow-up periods, limited choice of SSRIs, using different doses of 5-HT3 antagonists and SSRIs, using different rate of score changes for defining partial and complete response and remission, including only patients with moderate to severe OCD (not generalized to treatment-resistant OCD), not eliminating the effect of comorbidities, and not comparing the OCD subtypes. Plus, no clinical trial has investigated the effect of palonosetron on moderate to severe OCD.

We hope that further studies will investigate the effect of these groups of medications in multi-center trials under adequate conditions in more extended follow-up periods to help come up with a comprehensive action plan. We suggest that further trials investigate the effect of these medications in patients with OCD and comorbid disorders.

## Conclusions

Augmentation of 5-HT3 antagonists with SSRIs can be beneficial in treating moderate to severe OCD. They are safe and efficient, and can be used when the first-line treatment fails to achieve acceptable response. Although, to increase the certainty of these findings, further research is needed.

### Supplementary Information

Supplementary Information.

## Data Availability

The datasets generated and/or analysed during the current study are available from the corresponding author on reasonable request.

## Abbreviations

OCD: Obsessive–compulsive disorder
NMDA: *N*-Methyl-d-aspartate
SSRI: Selective serotonin reuptake inhibitor
DSM-5: Diagnostic and Statistical Manual of Mental Disorders
Y-BOCS: Yale-Brown obsessive compulsive scale
CBT: Cognitive-behavioral therapy
ERP: Exposure and response prevention
5-HT_3_: 5-Hydroxytryptamine receptor type 3
RCTs: Randomized-controlled trails
WHO: World Health Organization
GABA: γ-Aminobutyric acid
MD: Mean difference
CI: Confidence interval
SD: Standard deviation
CSTC: Cortico-striatal-thalamo-cortical
WFSBP: World Federation of Societies of Biological Psychiatry
NAC: *N*-Acetyl cysteine
TNF-α: Tumor necrosis factor-α
COX-2: Cyclooxygenase-2
PRISMA: Preferred Reporting Items for Systematic Reviews and Meta-Analyses for Protocols guidelines
RR: Risk ratio
