# Supplementary material for: Efficacy and safety of 5-hydroxytryptamine-3 (5-HT3) receptor antagonists in augmentation with selective serotonin reuptake inhibitors (SSRIs) in the treatment of moderate to severe obsessive–compulsive disorder: a systematic review and meta-analysis of randomized clinical trials
Source: Sci Rep. 2023 Nov 27;13:20837. doi: 10.1038/s41598-023-47931-x (PMC10682036; doi:10.1038/s41598-023-47931-x)
Supplement: Supplementary file 1 — Supplementary Information. [file 41598_2023_47931_MOESM1_ESM.pdf]

Topic as in PICOT format

P: Adult patients with obsessive-compulsive disorder as defined by the Diagnostic and Statistical Manual of Mental Disorders (DSM–5) (irrespective of age, gender, or race).

I: Using pharmaceutical agents that directly attenuate glutamatergic outflow 5-HT3 receptors as an adjunctive therapy.

C: Patients under treatment with SRIs. SRIs include clomipramine and selective serotonin reuptake inhibitors (SSRIs).

O: Y-BOCS score reduction.

T: All clinical trials investigating glutamatergic agents (according to the definitions in [I]) will be included, irrespective of randomization and blinding.

Search strategy

Databases

Journal articles

1. Web of Science
2. PubMed
3. Scopus
4. Cochrane library
5. Google Scholar

Keywords

P:

1. OCD[All fields]
2. Obsessive-compulsive[All fields]
3. obsess* [Title/Abstract]
4. compulsi*[Title/Abstract]
5. "Obsessive-Compulsive Disorder" [Mesh]
6. #1 OR #2 OR #3 OR #4 OR #5

I:

1. granisetron[All fields]
2. palonosetron[All fields]
3. ondansetron[All fields]

4. tropisetron[All fields]

5. Serotonin 5-HT3 Receptor Antagonists[MeSH Terms]

6. 5-HT3 receptor*[All fields]

7. 5ht3*[All fields]

8. 5-ht3*[All fields]

9. serotonin3 antagonist*[All fields]

10. #1 OR #2 OR #3 OR #4 OR #5 OR #6 OR #7 OR #8 OR #9

Combined search

1. #6 AND #10

Restrictions

No language, filter or date restriction

Specific issues:

[MeSH] only can be used in PubMed.
